# *CDKN2A* Determines Mesothelioma Cell Fate to EZH2 Inhibition

**DOI:** 10.3389/fonc.2021.678447

**Published:** 2021-07-01

**Authors:** Giulia Pinton, Zhuo Wang, Cecilia Balzano, Sara Missaglia, Daniela Tavian, Renzo Boldorini, Dean A. Fennell, Martin Griffin, Laura Moro

**Affiliations:** ^1^ Department of Pharmaceutical Sciences, University of Piemonte Orientale (UPO), Novara, Italy; ^2^ School of Life and Health Sciences, Aston University, Birmingham, United Kingdom; ^3^ Laboratory of Cellular Biochemistry and Molecular Biology, Centro di Ricerca in Biochimica E Nutrizione dello Sport (CRIBENS), Catholic University of the Sacred Heart, Milan, Italy; ^4^ Department of Health Science, University of Piemonte Orientale (UPO), Novara, Italy; ^5^ Leicester Cancer Research Centre, University of Leicester, Leicester, United Kingdom

**Keywords:** malignant pleural mesothelioma, EZH2 inhibitor, *CDKN2A*/p16^ink4a^, TG2, multicellular spheroids

## Abstract

Malignant pleural mesothelioma is an aggressive cancer, heterogeneous in its presentation and behaviour. Despite an increasing knowledge about molecular markers and their diagnostic and prognostic value, they are not used as much as they might be for treatment allocation. It has been recently reported that mesothelioma cells that lack BAP1 (BRCA1 Associated Protein) are sensitive to inhibition of the EZH2 (Enhancer of Zeste Homolog 2) histone methyltransferase. Since we observed strong H3K27me3 (histone H3 lysine 27 trimetylation) immunoreactivity in BAP1 wild-type mesothelioma biopsies, we decided to characterize *in vitro* the response/resistance of BAP1 wild-type mesothelioma cells to the EZH2 selective inhibitor, EPZ-6438. Here we demonstrate that BAP1 wild-type mesothelioma cells were rendered sensitive to EPZ-6438 upon SIRT1 (Sirtuin 1) silencing/inhibition or when cultured as multicellular spheroids, in which SIRT1 expression was lower compared to cells grown in monolayers. Notably, treatment of spheroids with EPZ-6438 abolished H3K27me3 and induced the expression of *CDKN2A* (Cyclin-Dependent Kinase Inhibitor 2A), causing cell growth arrest. EPZ-6438 treatment also resulted in a rapid and sustained induction of the genes encoding HIF2α (Hypoxia Inducible Factor 2α), TG2 (Transglutaminase 2) and IL-6 (Interleukin 6). Loss of *CDKN2* is a common event in mesothelioma. *CDKN2A* silencing in combination with EPZ-6438 treatment induced apoptotic death in mesothelioma spheroids. In a *CDKN2A* wild-type setting apoptosis was induced by combining EPZ-6438 with 1-155, a TG2 selective and irreversible inhibitor. In conclusion, our data suggests that the expression of *CDKN2A* predicts cell fate in response to EZH2 inhibition and could potentially stratify tumors likely to undergo apoptosis.

## Introduction

Malignant pleural mesothelioma (MPM) is a lethal cancer that originates from the mesothelial cells aligning the pleura (1). The causal association of MPM with asbestos exposure is well established and supported by epidemiological and toxicological studies (2, 3). Although asbestos has been banned in numerous countries, it continues to be used worldwide, and a rise in the MPM global incidence is predicted (4).

The most commonly used chemotherapy drugs for treating MPM include pemetrexed with cisplatin or carboplatin (5). The high number of non-responders to chemotherapy, as well as the frequent recurrences of the disease (6, 7), suggests the presence of drug-resistant clones within the tumor. Given that MPM develops over many years and its growth rate is in most cases quite low, it is highly likely that it has profound heterogeneity, which makes it challenging to eradicate (8).

MPM intra- and inter-tumor heterogeneity manifests with a morphological spectrum, ranging from epithelioid to sarcomatoid tumors, with the biphasic subtype containing a combination of both components (9). Besides histological diversity, an increasing number of publications highlight the importance of genetic and epigenetic intra-tumor heterogeneity for MPM therapeutic resistance (10–13). Genomic interrogation has revealed extensive interpatient heterogeneity with frequent tumor suppressor inactivation being a dominant feature of the mutation landscape mediated by multiple mechanisms, which include single nucleotide variation, copy number losses, gene fusions and splicing alterations (14, 15). Loss or mutation of BAP1 (BRCA1 Associated Protein 1) coding gene is recurrently identified in MPM and translates into nuclear negativity for BAP1 expression (16). Levine’s group has reported that *BAP1* inactivation leads to upregulation and dependence on the chromatin modifying Polycomb Repressive Complex 2 (PRC2), comprising the methyltransferase EZH2 (Enhancer of Zeste Homolog 2), which trimethylates lysine 27 on histone H3 (H3K27me3) (17). Besides transcriptional regulation, post-translational modifications of EZH2, including phosphorylation (18), acetylation (19), ubiquitination (20), sumoylation (21) and GlcNAcylation (22), have also been found to be important for its expression and silencing function on target genes.

Another common genetic alteration in MPM is the homozygous deletion of the 9p21 locus, within a cluster of genes that includes *CDKN2A* (cyclin-dependent kinase inhibitor 2A) encoding p16^ink4a^ (10, 23, 24). Heterozygous deletion of *CDKN2A* is also commonly observed, sometimes at higher levels than homozygous deletion, however, few reports have addressed its role in MPM (25–27). Furthermore, a discrepancy between p16^ink4a^ protein expression and gene deletion suggests that epigenetic mechanisms play a role in its regulation (28). Indeed, H3K27me3-dependent repression of *CDKN2A* transcription is a common feature of many tumors (29, 30).

H3K27 methylation can also be influenced by hypoxia (31). We have described that HIF-2α (Hypoxia Inducible Factor 2α) induces the expression of the H3K27 demethylase KDM6B and reduces the H3K27me3 repressive mark in MPM cells exposed to chronic hypoxia (32). Furthermore, we have described that HIF-2α-mediates the induction of *TGM2* and the increase of TG2 (Transglutaminase 2) activity (33). TG2 is a multifunctional enzyme that exhibits crosslinking, GTPase, cell adhesion, protein disulfide isomerase, kinase, and scaffold activities (34). TG2 expression and downstream IL-6 (Interleukin-6) production have been profoundly correlated with primary tumor growth, peritoneal spreading, distant metastasis and resistance to standard cytotoxic agents (35, 36). We have demonstrated that TG2 inhibition or *TGM2* silencing causes, under hypoxic conditions, a significant reduction of MPM cell viability (33).

Here we describe strong H3K27me3 immunoreactivity in tumor tissue samples obtained from patients diagnosed with BAP1 wild-type MPM. Moreover, we report that the treatment of MPM multicellular spheroids with an EZH2 selective inhibitor induces *CDKN2A* expression and cell cycle arrest. Finally, we describe the involvement of p16^ink4a^ and TG2 in the control of MPM apoptotic cell death in response to EZH2 inhibition.

## Materials and Methods

### Reagents and Antibodies

The polyclonal antibodies specific for histone H3 trimethyl lysine 27 (H3K27me3), histone H3, SIRT1, KDM6B and the monoclonal antibodies specific for Poly (ADP-ribose) polymerase 1 (PARP1), acetyl-lysine, HIF2α, BAP1 and α-tubulin were purchased from Santa Cruz Biotechnology (Santa Cruz CA, USA). Polyclonal antibody anti-p16^ink4a^ was from Cell Signaling Technology (Leiden, The Netherlands). Polyclonal antibody specific for EZH2 was from Active Motif (La Hulpe, Belgium). Anti-mouse and anti-rabbit IgG peroxidase or FITC conjugated antibodies and chemical reagents were from Sigma-Aldrich (St. Louis, MO, USA). ECL, nitrocellulose membranes and protein assay kit were from Bio-Rad (Hercules, CA, USA). Culture media, sera, antibiotics, LipofectAMINE transfection reagent and the monoclonal antibody specific for TG2 (TG100) were from Thermo Fisher (Waltham, MA, USA). EX-527 and the EZH2-selective inhibitor, EPZ-6438, were from Selleckchem (Houston, TX, USA). The highly selective TG2 inhibitor, 1-155 (cell-permeable) was designed and synthesized as previously documented (37, 38).

### Immunohistochemistry on Formalin-Fixed Paraffin-Embedded Tissue Specimens

BAP1 and histone H3 trimethyl lysine 27 (H3K27me3) expression were evaluated by immunohistochemistry (IHC) in eight biphasic MPM human biopsies using a BenchMark standard automated immunostainer (Ventana Medical System, Tucson, AZ, USA). Specific primary antibodies against the anti-human BAP1 and the polyclonal antibodies specific for H3K27me3 were used. BAP1 and H3K27me3 were considered positive when a weak-to-strong nuclear positivity was shown. Negative controls were obtained by replacing the primary antibody by PBS. Non-neoplastic cells, such as vascular endothelium or inflammatory cells, were considered as internal positive controls. Slides were counterstained with hematoxylin.

### Cell Cultures and Transfection

The biphasic MPM derived MSTO-211H cell lines was obtained from the Istituto Scientifico Tumori (IST) Cell-bank, Genoa, Italy; the epithelioid MPM BR95 cell line was kindly provided by Prof. Osella D. (University of Piemonte Orientale, Alessandria, Italy). Cells were grown in standard conditions in RPMI medium supplemented with 10% FBS, 100 μg/ml streptomycin and 10 μg/ml penicillin at 37°C in a humidified environment containing 5% CO_2_. Mycoplasma infection was excluded by the use of Mycoplasma PlusTM PCR Primer Set kit from Stratagene (La Jolla, CA, USA). Cells grown to 80% confluence in tissue culture dishes were transiently transfected with negative control or specific siRNAs from Qiagen (Hilden, Germany) using LipofectAMINE reagent as described by the manufacturer. To obtain cell number and viability information following treatments, cell were trypsinized and stained with Trypan blue; the number of cells considered viable (unstained cells) was counted in a Bürker chamber within 5 min after staining.

### Multicellular Spheroids

Multicellular spheroids were generated in non-adsorbent round-bottomed 96-well plates, as previously described (31). The 96-well plates were coated with a 1:24 dilution of polyHEMA (120 mg/ml) in 95% ethanol and dried at 37°C for 24 h. Before use, plates were sterilized by UV light for 30 min. For generation of multicellular spheroids, 1 × 10^4^ cells were added into each well of polyHEMA-coated 96-well plate and placed in a 37°C humidified incubator with 5% CO_2_. Every 24 h, 50% of supernatant was replaced with fresh medium ± 10 μM EPZ-6438 and/or 5 μM 1-155 inhibitors. For cell dissemination, spheroids treated for 48 h with or without EPZ-6438, were transferred to flat-bottomed culture dishes and incubated in complete medium for additional 24 h. Migration area was observed under the microscope.

### Immunofluorescence Staining

Cell spheroids were let to adhere for 1/2 h to poly-L-lysine coated glass slides and then fixed in 4% paraformaldehyde, permeabilized with 0.5% Triton X-100 in PBS and blocked in 3% BSA/PBS 10% FBS. The primary antibody (mouse anti-H3K27me3 1:100) was incubated for 2 h at 4°C. The fluorescent secondary antibody (rabbit anti-mouse IgG antibody conjugated with fluorescein isothiocyanate (FITC); 1:100) was incubated for 1 h at 4°C. Fluorescent images were captured using a Leica MB5000B microscope equipped with a DFC480 R2 digital camera and a Leica Application Suite (LAS) software.

### Cell Cycle Analysis

For cell cycle/apoptosis analysis, 5 × 10^5^ cells were silenced for *SIRT1* and treated or not with EPZ-6438 for 48 h at 37°C in a 5% CO_2_ atmosphere. After incubation, detached and suspended cells were harvested in complete RPMI and centrifuged at 500×*g* for 10 min. Pellets were washed with PBS, pH 7.4, in ice-cold 75% ethanol at 4°C, treated with 100 mg/ml RNAse A for 1 h at 37°C, stained with 25 μg/ml propidium iodide and finally analyzed by using a Bio-Rad S3e Cell Sorter (Hercules, CA, USA) and the Modfit software (Verity Software House, Topsham, ME, USA).

### Cell Lysis and Immunoblot

Cells were extracted with 1% NP-40 lysis buffer (50 mM Tris–HCl pH 8.5 containing 1% NP-40, 150 mM NaCl, 10 mM EDTA, 10 mM NaF, 10 mM Na_4_P_2_O_7_ and 0.4 mM Na_3_VO_4_) with freshly added protease inhibitors (10 μg/ml leupeptin, 4 μg/ml pepstatin and 0.1 Unit/ml aprotinin). Lysates were centrifuged at 13.000×*g* for 10 min at 4°C and the supernatants were collected and assayed for protein concentration with the Bradford assay method (Bio-Rad). Histones were acid extracted from nuclei with 0.4 N HCl and precipitated with trichloroacetic acid (TCA), followed by washing with ice-cold acetone containing 0.006% HCl, and then with pure ice-cold acetone. The resulting pellets were air-dried, dissolved in a minimal volume of sterile distilled water and the protein concentration was determined.

Proteins were separated by SDS-PAGE under reducing conditions. Following SDS-PAGE, proteins were transferred to nitrocellulose, reacted with specific antibodies and then detected with peroxidase-conjugate secondary antibodies and chemioluminescent ECL reagent. Digital images were taken with the Bio-Rad ChemiDoc™ Touch Imaging System and quantified using Bio-Rad Image Lab 5.2.1.

### RNA Isolation and Real-Time PCR

Total RNA was extracted using the guanidinium thiocyanate method. Starting from equal amounts of RNA, cDNA used as template for amplification in the real-time PCR (5 µg), was synthesized by the reverse transcription reaction using RevertAid Minus First Strand cDNA Synthesis Kit from Fermentas-Thermo Scientific (Burlington, ON, Canada), using random hexamers as primers, according to the manufacturer’s instructions. Some 20 ng of cDNA were used to perform RT-PCR amplification of mRNA. The real-time reverse transcription-PCR was performed using the double-stranded DNA-binding dye SYBR Green PCR Master Mix (Fermentas-Thermo Scientific) on an ABI GeneAmp 7000 Sequence Detection System machine, as described by the manufacturer. The instrument, for each gene tested, obtained graphical Cycle threshold (Ct) values automatically. Triplicate reactions were performed for each marker and the melting curves were constructed using Dissociation Curves Software (Applied Biosystems, Foster City, CA, USA), to ensure that only a single product was amplified.

### Statistical Analysis

Statistical evaluation of the differential analysis was performed by one-way ANOVA and Student’s t-test.

## Results

### BAP1 Wild-Type Mesotheliomas Exhibit H3K27me3 Immunoreactivity

BAP1 is inactive in 40–60% of MPM patients (10, 14). Recent evidence suggesting the potential efficacy of EZH2 inhibition in preclinical models of BAP1 loss led to a phase II clinical trial evaluating the EZH2 inhibitor Tazemetostat (EPZ-6438) in MPM patients with inactive BAP1 (NCT02860286).

**Figure 1:**
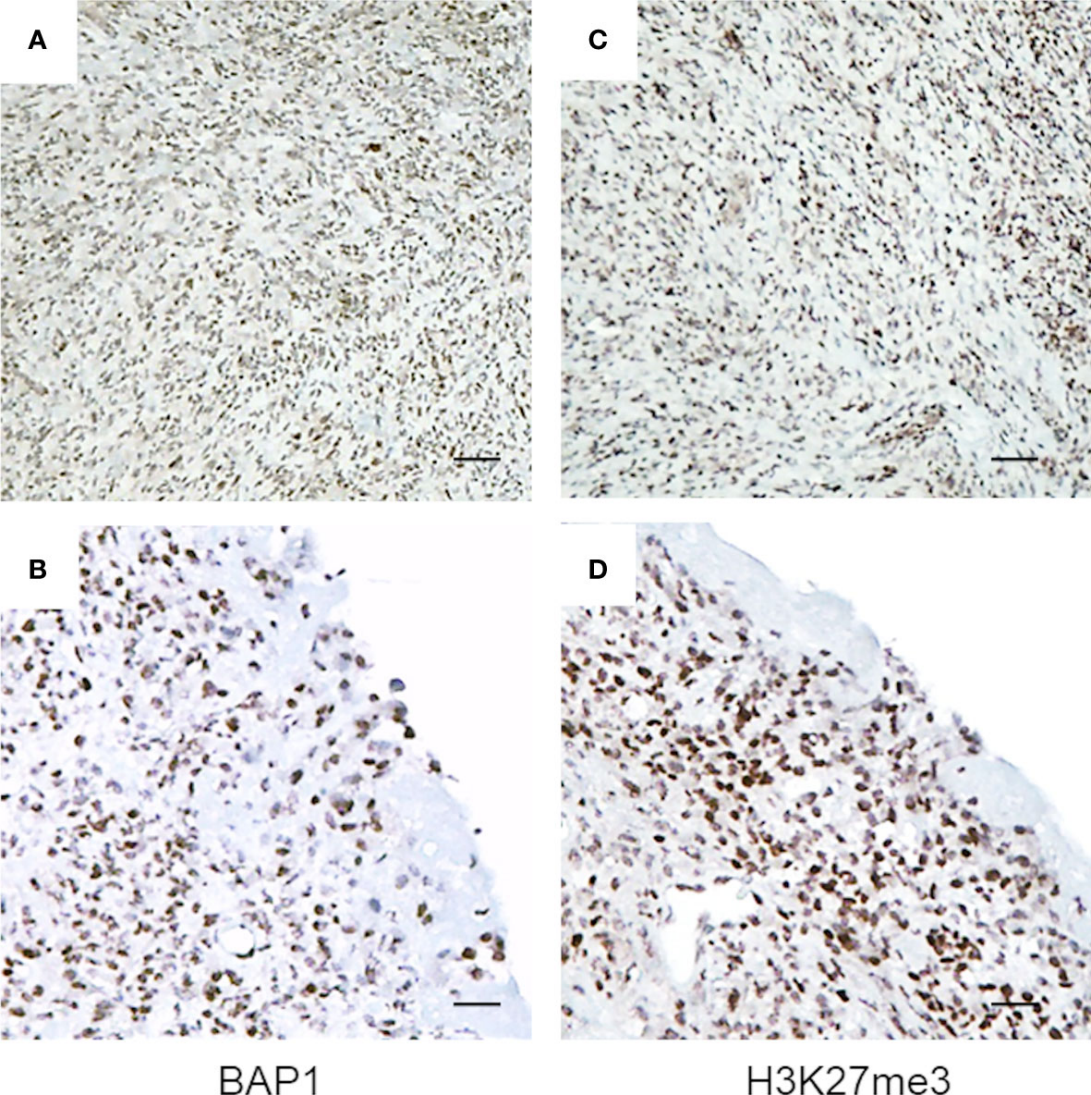
BAP1 wild-type MPM tumor biopsies exhibit high immunoreactivity for H3K27me3. Representative images of immunostaining. Byphasic MPM tissue samples were stained for BAP1 **(A, B)** and H3K27me3 **(C, D)**. Images were captured at magnification ×40 **(A, C)** and ×200 **(B, D)** using light microscopy. Scale bar = 100 μM.

Here, we examined the H3K27me3 status in biopsies obtained at diagnosis from a cohort of BAP1 positive, biphasic MPM, using immunohistochemistry (IHC). All tissues examined showed strong BAP1 and H3K27me3 nuclear staining in the majority of tumor cells (3+ in >67%) (**Figures 1A–D** and **Figures S1A–F**). A minority of cells displayed a reduced H3K27me3 nuclear staining, however, further and deeper investigation is needed in order to associate significant differences in intensity with the epithelioid or the sarcomatoid component of the tumor. Strong immunoreactivity for H3K27me3 was also observed in the non-neoplastic infiltrating inflammatory cells (**Figures S1E, F**).

### Inhibition of EZH2 Activity Exerts Anti-Proliferative Effect in BAP1 Wild-Type MPM Spheroids

Based on IHC results, we decided to use the MSTO-211H cell line, established from the pleural effusion of a patient with biphasic mesothelioma, as a model to better characterize *in vitro* the response of BAP1 wild-type MPM to EPZ-6438, a potent and selective inhibitor of the H3K27 methyltransferase EZH2.

**Figure 2:**
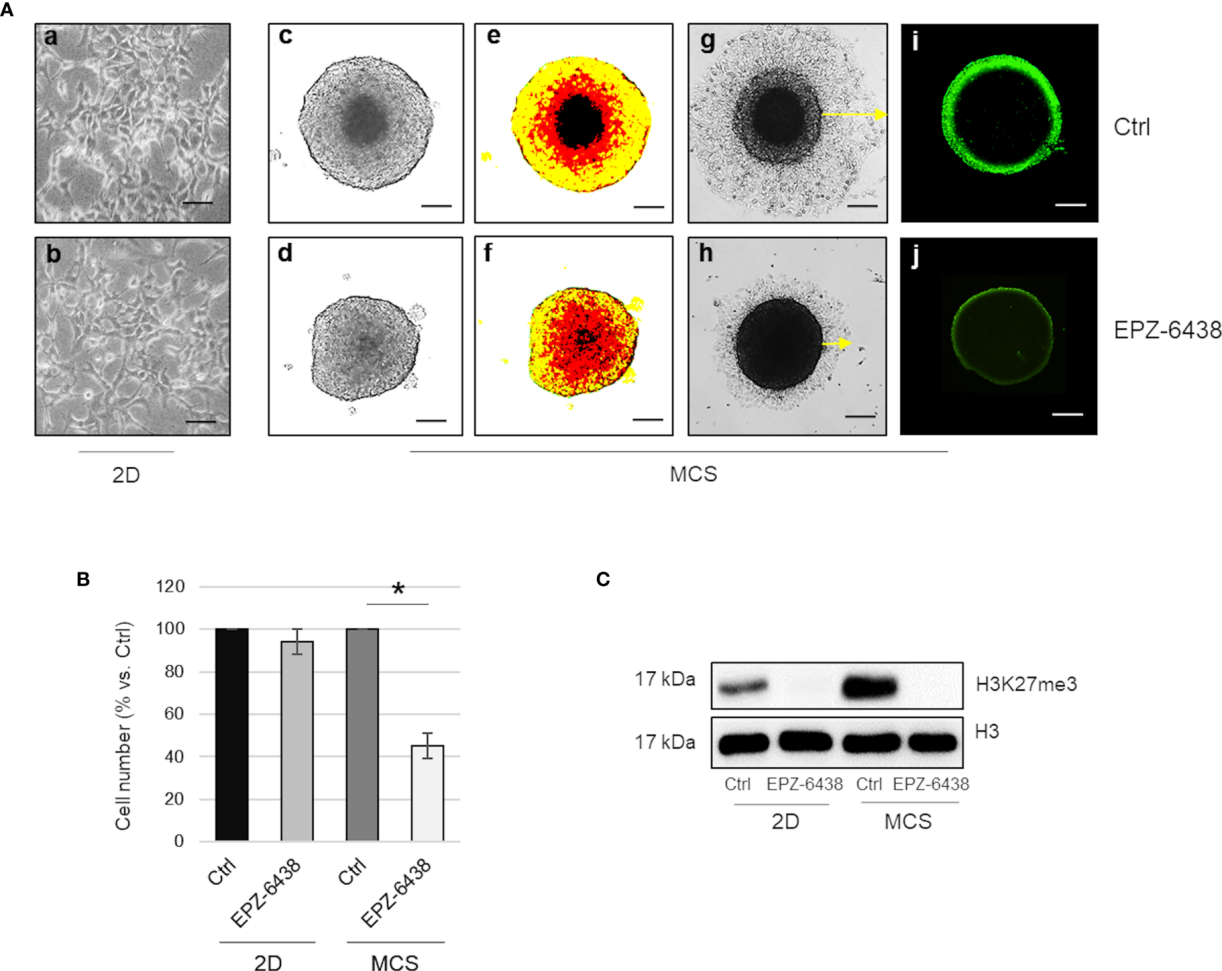
EZH2 inhibition displays an antiproliferative effect in BAP1 wild-type MPM MCS. **(A)** Representative phase contrast images (×40 magnification) of MSTO-211H cultured as monolayer **(a, b)** or MCS (1 of 12) **(c, d)** with relative pseudo-color images **(e, f)**. Representative phase contrast images of dissemination of MSTO-211H MCS (one of 12) after 24 h re-adhesion to flat-bottomed plates, post 48 h treatment in the presence or absence of EPZ-6438 **(g, h)**. H3K27me3 immunofluorescence image of MSTO-211H MCS treated for 48 h, with or without EPZ-6438 **(i, j)**. Scale bar = 100 μM. **(B)** Bar graph shows the percentage of viability of MSTO-211H cells cultured as monolayer or MCS ± 48 h treatment with EPZ-6438. Each bar represents mean of three independent experiments ± s.d., *p ≤0.05. **(C)** Representative Western blot analysis of H3K27me3 in MSTO-211H cultured in 2D or as MCS ± 48 h treatment with EPZ-6438. histone H3 was used as loading control.

We first report, as described by LaFave et al. (17), that inhibition of the EZH2 methyltransferase activity, by EPZ-6438, did not affect MSTO-211H cell viability, when cultured as two-dimensional (2D) monolayer (**Figures 2A, a, b, B**). Differently, we observed that EPZ-6438 treatment significantly reduced the size and modified the architecture of MSTO-211H multicellular spheroids (MCS), as evidenced by bright field and pseudo-color images (**Figures 2A, c–f**), and reduced cell dissemination after re-adhesion of MCS to flat-bottomed plates (**Figures 2A, g, h**). Cell counts confirmed that the reduction in size of EPZ-6438 treated MCS was due to a lower number of cells and not to an increase in cell packing density (**Figure 2B**). Immunofluorescence images in **Figures 2A(i, j)** show that H3K27me3 was spatially restricted to the external proliferating region of the spheroids and was completely abolished by EPZ-6438 treatment. In agreement, we documented high expression of the H3K27 demethylase KDM6B in the internal hypoxic zone of the spheroids, enriched in HIF2α expression (**Figure S2**) (31). Western-blot analysis confirmed an increase in H3K27 methylation in MSTO-211H MCS *versus* 2D culture that was completely abolished by EPZ-6438 treatment (**Figure 2C**).

### SIRT1 Inhibition Confers Sensitivity to EZH2 Inhibition in BAP1 Wild-Type MPM Cells

**Figure 3:**
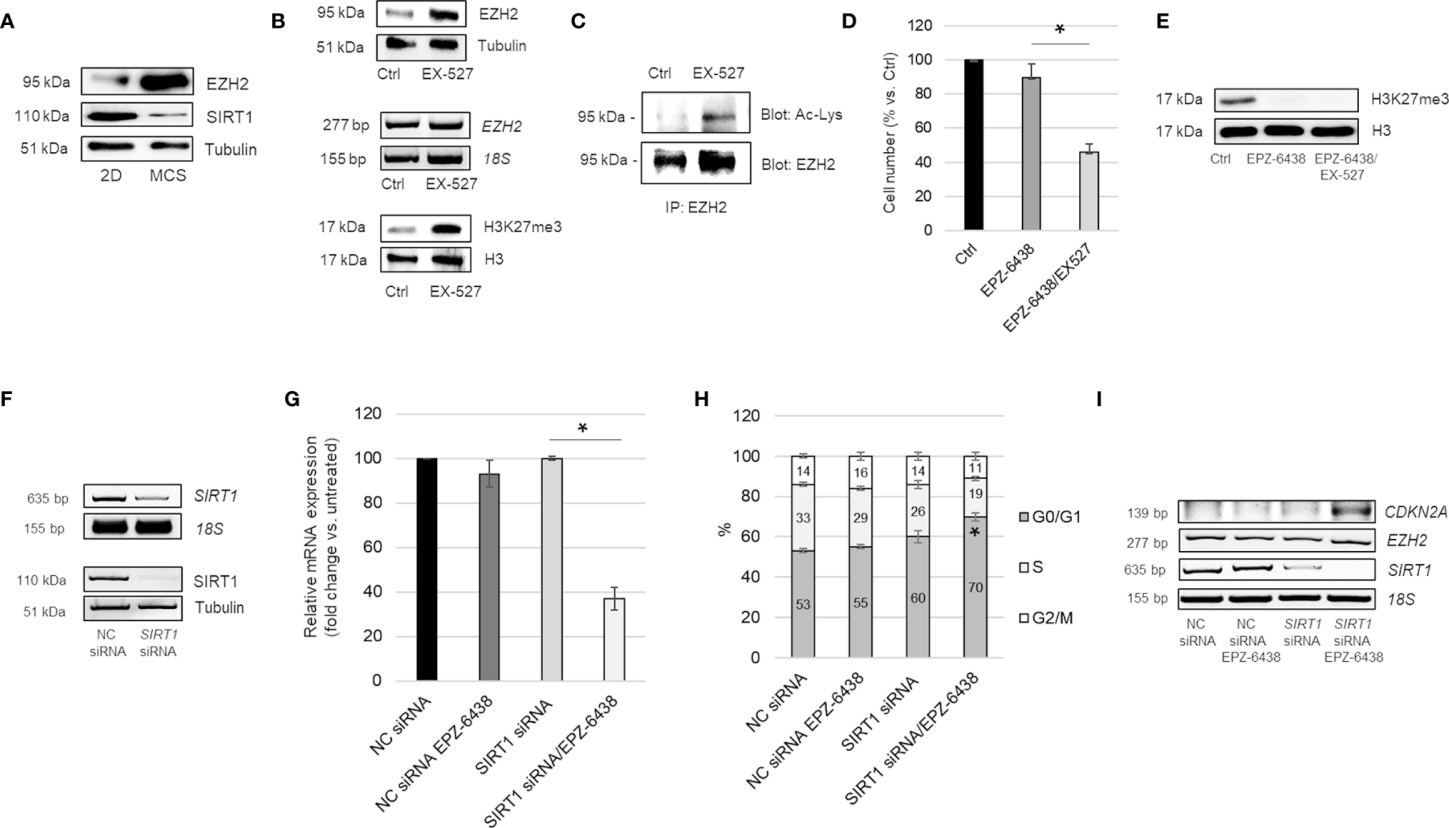
SIRT1 inhibition increases EZH2 expression, acetylation and activity. **(A)** Representative Western blot analysis of EZH2 and SIRT1 in MSTO-211H cells cultured 48 h as monolayer or as MCS. Tubulin was used as loading control. **(B)** Representative Western blot analysis and RT-PCR analysis of EZH2 expression and of H3K27 trimethylation (H3K27me3) in MSTO-211H cells cultured as monolayer ± 48 h EX527 treatment. Tubulin and histone H3 were used as loading control and 18S rRNA as housekeeping gene. **(C)** Immunoprecipitation of EZH2 from lysates of MSTO-211H cells ± EX527 48 h treatment. Lysine acetylation and EZH2 were detected by Western blot analysis with the respective antibodies (AcLys and EZH2). **(D)** Bar graph shows the percentage of viability of MSTO-211H cells cultured as monolayer ± 48 h treatment with EPZ-6438 or EPZ-6438/EX527 combination. Each bar represents mean of three independent experiments ± s.d., *p ≤ 0.05. **(E)** Representative Western blot analysis of H3K27me3 in MSTO-211H cells treated 48 h with EPZ-6438 or EPZ-6438/EX527 combination. Histone H3 was used as loading control. **(F)** Representative Western blot analysis and RT-PCR analysis of SIRT1 expression in MSTO-211H cells transfected with negative control (NC) or *SIRT1* specific siRNAs (*SIRT1* siRNA). Tubulin was used as loading control and 18S rRNA as housekeeping gene. **(G)** Bar graph shows the percentage of viability of MSTO-211H cells transfected with negative control (NC) or *SIRT1* specific siRNAs (*SIRT1* siRNA) after 48 h treatment ± EPZ-6438. Each bar represents mean of three independent experiments ± s.d., *p ≤ 0.05. **(H)** Bar graph representing the percentage of cells in G0/G1, S and G2 phase of MSTO-211H cells transfected with negative control (NC) or *SIRT1* specific siRNAs (*SIRT1* siRNA) and treated or not, 48 h, with EPZ-6438. Bars represent the means of three measurements ± s.d., *p < 0.05. **(I)** Representative quantitative RT-PCR analysis of *CDKN2A*, *EZH2* and *SIRT1 in* MSTO-211H cells transfected with negative control (NC) or *SIRT1* specific siRNAs (*SIRT1* siRNA) and treated 48 h ± EPZ-6438. 18S rRNA was used as housekeeping gene.

It has been reported that depletion of the NAD-dependent deacetylase SIRT1 increases EZH2 protein acetylation, stability and its repressive effect on target genes (19). In our previous studies, we demonstrated lactate-mediated SIRT1 downregulation, in MPM cells cultured as MCS compared to monolayer (39). Here, along with SIRT1 downregulation, we observed increased expression of EZH2 in MSTO-211H MCS (**Figure 3A**). To evaluate the regulatory role of SIRT1 on EZH2 expression and activity in BAP1 wild-type MPM, we treated MSTO-211H cells in 2D with the SIRT1 selective inhibitor, EX-527, alone or in combination with EPZ-6438. In MSTO-211H cells treated with EX-527, we demonstrated the expression of EZH2 protein significantly increased, whereas EZH2 mRNA levels remained stable (**Figure 3B**). In this experimental condition, we demonstrated, by immunoprecipitation experiments, an increase in EZH2 acetylation (**Figure 3C**). In accordance with increased EZH2 protein expression and acetylation, we observed increased H3K27me3 level in EX-257 treated cells (**Figure 3B**). The combined treatment of EPZ-6438 and EX-527 resulted in a significant reduction in the MSTO-211H growth rate (**Figure 3D**). H3K27 demethylation was confirmed by Western blot analysis (**Figure 3E**).

To confirm the role of SIRT1 in cell response to EPZ-6438 treatment, we transfected MSTO-211H cells with specific siRNAs and evaluated the effect on cell proliferation. SIRT1 silencing was confirmed by RT-PCR and Western blot analyses (**Figure 3F**). Comparable EPZ-6438 anti-proliferative effect, accompanied by a G1 phase cell cycle arrest, was observed in SIRT1 silenced cells (**Figures 3G, H**). We observed that EPZ-6438 treatment induced the expression of *CDKN2A*, encoding for the cell cycle inhibitor p16^ink4a^, in *SIRT1* silenced cells cultured as monolayer (**Figure 3I**), but not in cells transfected with negative control siRNA.

### EZH2 Inhibition Mediates Strong Induction of *CDKN2A* in MPM Spheroids

**Figure 4:**
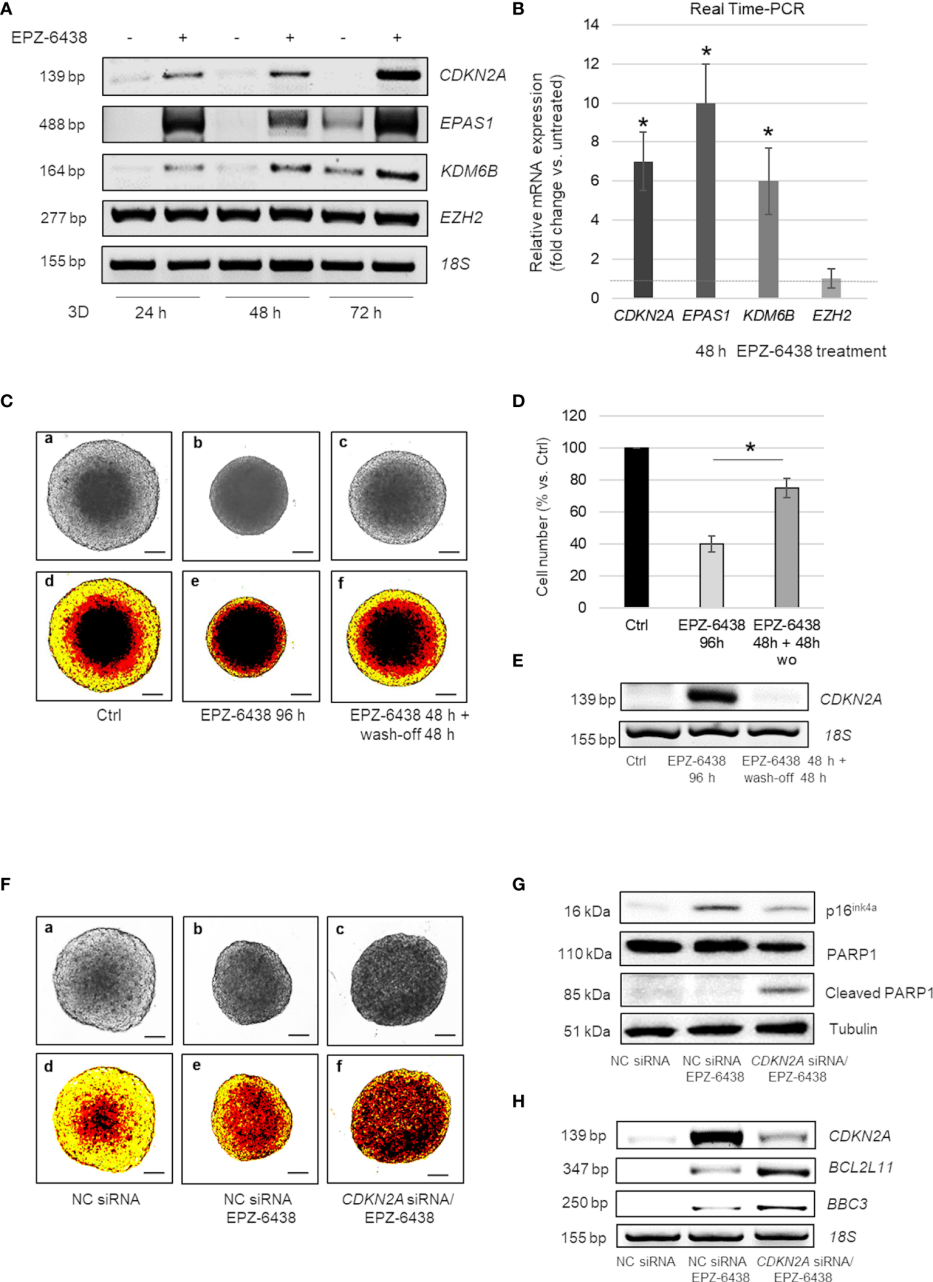
EZH2 inhibition induces cell cycle arrest in *CDKN2A^+^* or apoptosis in *CDKN2A^-^* MPM MCS **(A)** Representative RT-PCR analysis of *CDKN2A, EPAS1, KDM6B* and *EZH2 in* MSTO-211H MCS treated or not 24, 48 and 72 h with EPZ-6438. **(B)** *CDKN2A, EPAS1, KDM6B and EZH2* mRNA expression (fold increase over Ctrl) after 48 h treatment with EPZ-6438 of MSTO-211H MCS confirmed by Real time PCR (qPCR). 18S rRNA was used as housekeeping gene. **(C)** Representative phase contrast images (×40 magnification) **(a–c)** and relative pseudo-color images **(d–f)** of MSTO-211H MCS treated ± EPZ-6438 for 96 h or treated for 48 h with EPZ-6438 and, after wash-off, grown for additional 48 h in normal medium. **(D)** Bar graph shows the percentage of viability of MSTO-211H MCS treated 96 h with EPZ-6438 or treated for 48 h with EPZ-6438 and, after wash-off, grown for additional 48 h in normal medium. Each bar represents mean of three independent experiments ± s.d., *p ≤0.05. **(E)** Representative quantitative RT-PCR analysis of *CDKN2A in* MSTO-211H MCS treated as described above. 18S rRNA was used as housekeeping gene. **(F)** Representative phase contrast images (×40 magnification) (one of 12) **(a–c)** and relative pseudo-color images **(d–f)** of MSTO-211H MCS transfected with negative control (NC) siRNAs or *CDKN2A* specific siRNAs (*CDKN2A* siRNA) and treated ± EPZ-6438, 48 h. Scale bar = 100 μM. **(G)** Representative Western blot analysis of p16^ink4a^, PARP1 and cleaved PARP1 in MSTO-211H MCS transfected with negative control (NC) siRNAs or *CDKN2A* specific siRNAs (*CDKN2A* siRNA) and treated with or without EPZ-6438 for 48 h. Tubulin was used as loading control. **(H)** Representative RT-PCR analyses of *CDKN2A*, *BCL2L11 and BBC3 in* MSTO-211H MCS transfected with negative control (NC) siRNAs or *CDKN2A* specific siRNAs (*CDKN2A* siRNA) and treated, 48 h, with EPZ-6438. 18S rRNA was used as housekeeping gene.

Notably, in EPZ-6438 treated MSTO-211H MCS, we observed a strong induction of *CDKN2A* within 24 h, with a gradual increase at 48 and 72 h (**Figures 4A, B**). Comparable results were observed in the *BAP1/CDKN2A* wild-type BR95 MPM cells, cultured as MCS (**Figure S3**).

Moreover, here we show that treatment with EPZ-6438 strongly induced the expression of genes encoding HIF2α and KDM6B, starting from 24 h of treatment (**Figures 4A, B**). In untreated MCS the expression of these genes increased starting from 72 h of culture, as we had previously described (31).

To determine whether EZH2 inhibition induced a temporary cell cycle arrest or a permanent exit from cycling, MSTO-211H MCS were treated 48 h with EPZ-6438, followed by its wash-off, and continued growth in normal culture medium for an additional 48 h. Interestingly, the cell growth and architecture of MSTO-211H MCS (**Figures 4C, a–f, D**) along with *CDKN2A* expression (**Figure 4E**) returned back to control levels after 48 h of EPZ-6438 removal.

### EZH2 Inhibition Induces Apoptosis in *CDKN2A* Silenced MPM Cells

As homozygous and heterozygous deletions of the 9p21 locus, encompassing *CDKN2A*, are frequent in MPM, we hypothesized that p16^ink4a^ induction was responsible for the arrest in cell growth observed in MCS treated with EPZ-6438 and that cells deleted for this gene could be insensitive to EZH2 inhibition. We next generated MCS from *CDKN2A* silenced MSTO-211H cells to counteract its induced expression in response to EPZ-6438 treatment. Different to our hypothesis, EPZ-6438 treatment induced apoptosis in *CDKN2A* silenced MSTO-211H MCS, as demonstrated by bright field (**Figures 4F, a–c**) and pseudo-color microscope images (**Figures 4F, d–f)**, PARP1 cleavage (**Figure 4H**), *BCL2L11* (encoding BIM) and *BBC3* (encoding PUMA) induction (**Figure 4F**). *CDKN2A* silencing was confirmed by Western blot (**Figure 4G**) and RT-PCR (**Figure 4H**) analysis.

### Dual Inhibition of EZH2 and TG2 Exhibits Proapoptotic Synergy in *CDKN2A* Wild-Type MPM Cells

**Figure 5:**
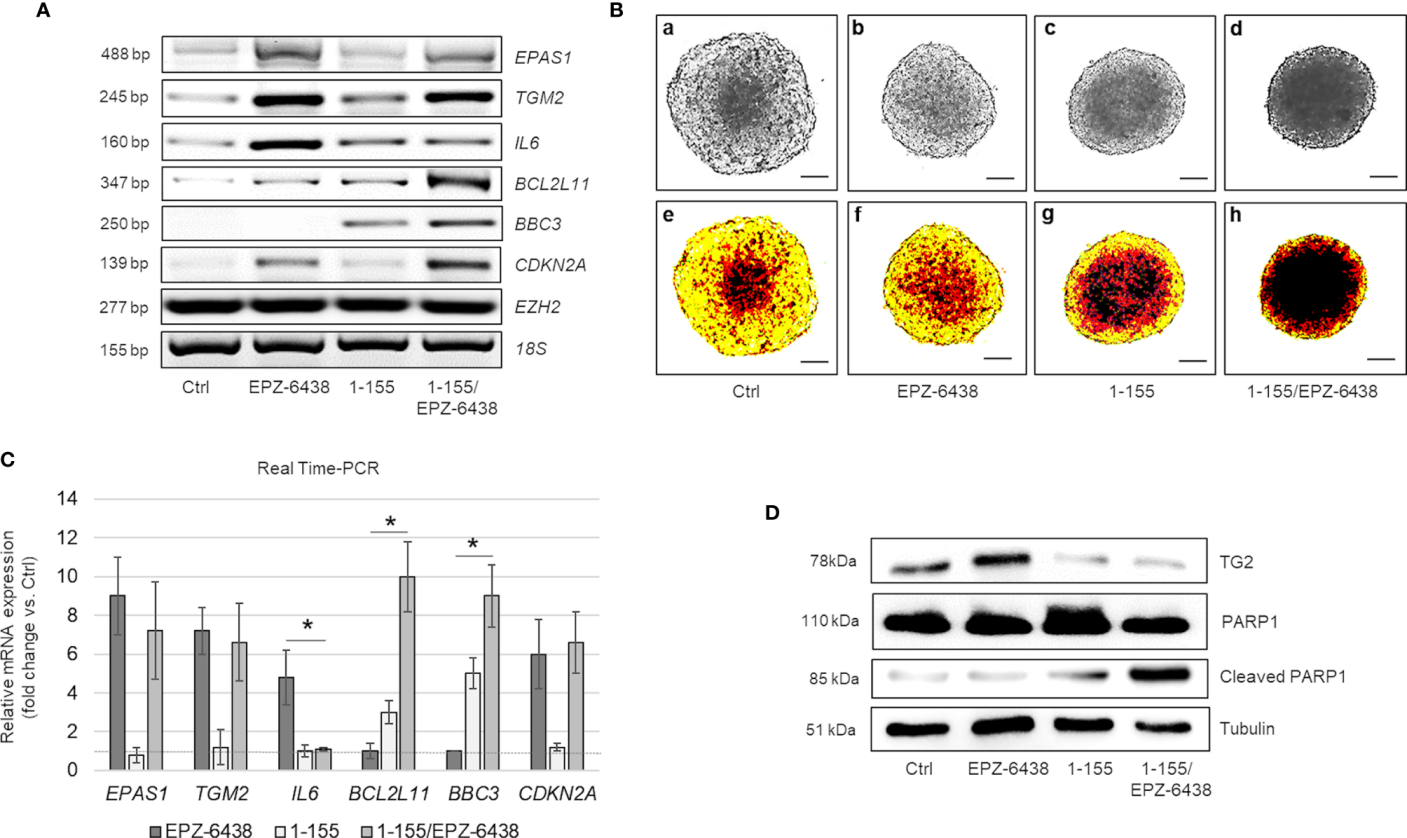
Dual inhibition of TG2 and EZH2 exhibits pro-apoptotic synergy in MPM MCS. **(A)** Representative RT-PCR of *EPAS1, TGM2, IL6, BCL2L11, BBC3, CDKN2A*, and *EZH2* expression in MSTO-211H MCS untreated or treated 48 h with EPZ-6438 and 1-155 as single agents or in combination. 18S rRNA was used as housekeeping gene. **(B)** Representative phase contrast images (×40 magnification) (one of 12) **(a–d)** and relative pseudo-color images **(e–h)** of MSTO-211H MCS untreated or treated 48 h with EPZ-6438 and 1-155 as single agents or in combination. Scale bar = 100 μM. **(C)** Real Time PCR (qPCR) analysis of *EPAS1, TGM2, IL6, BCL2L11, BBC3* and *CDKN2A* expression in MSTO-211H MCS untreated or treated 48 h with EPZ-6438 and 1-155 as single agents or in combination. 18S rRNA was used as housekeeping gene. Each bar represents mean of three independent experiments ± s.d., *p ≤ 0.05. **(D)** Representative Western blot analysis of TG2, PARP1 and cleaved PARP1 in MSTO-211H MCS untreated or treated 48 h with EPZ-6438 and 1-155 as single agents or in combination. Tubulin was used as loading control.

Looking for a potential druggable target in *CDKN2A* expressing MPM MCS, we tested whether EZH2 inhibition, by inducing rapid HIF2 expression, could also lead to increased TG2 expression. We have recently reported that HIF2 controls *TGM2* expression and that TG2 activity is essential for MPM cell survival under prolonged hypoxic condition. As shown in **Figures 5A, C**, EPZ-6438 induced the expression of *EPAS1*,*TGM2* and its target *IL-6* at 48 h treatment in MSTO-211H and in BR95 MCS (**Figures S3C, D**).

In order to test the importance of TG2 induction in preventing apoptosis, we treated MSTO-211H cells, cultured as MCS, with EPZ-6438 and the cell permeable TG2 selective inhibitor, 1-155, as single agents or in combination, for 48 h. As documented by bright field (**Figures 5B, a–d**) and pseudo-color microscope images (**Figures 5B, e–h**) the two drugs synergized in reducing MCS size. By Western-blot analysis, we observed a slow induction of TG2 expression in response to EPZ-6438 treatment and, as expected, accelerated breakdown in response to 1-155 treatment (**Figure 5D**). The EPZ-6438/1-155 combined treatment induced apoptosis as documented by increased PARP1 cleavage (**Figure 5D**), *BCL2L11* and *BBC3* expression, as documented by RT-PCR (**Figure 5A**) and Real Time PCR (**Figure 5C**), 1-155 treatment did not modify the EPZ-6438 induced expression of *CDKN2A, EPAS1* and *TGM2*, but significantly reduced the expression of the gene encoding IL-6 (**Figures 5A, C**).

## Discussion

MPM is known to show intrinsic therapy resistances and is so far incurable. Therefore, establishment of new therapies for this disease could be a valuable addition to current treatment options.

The high number of non-responders to chemotherapy (6), as well as the frequent recurrences of the disease (7), suggests a high degree of genetic heterogeneity within individual tumors. In concert with genetic variation, dynamic regulation of the epigenetic state may have important consequences for tumor plasticity and biology. Recent work has implicated mutation and/or dysregulated expression of histone lysine methyltransferases (KMTs) and demethylases (KDMs) in cancer (40).

Among KMTs, EZH2 has been reported to be overexpressed in various cancers, including MPM, and particularly to be associated with aggressiveness and poor prognosis. Pharmacological inhibition of EZH2 by 3-Deazanplanocin A (DZNep), an S-adenosylhomocysteine (SAH) hydrolase inhibitor, has been shown to effectively inhibit the growth of MPM cells (41, 42). A phase II clinical trial (NCT02860286) of Tazemetostat (EPZ-6438), a potent and selective EZH2 inhibitor, in MPM with inactive BAP1 has shown promising results (43).

However, BAP1 inactivation and H3K27me3 positivity in tissues may be sufficient, but not essential to predict response to EZH2 inhibition. Here we uncovered an antiproliferative activity of EPZ-6438 in BAP1 wild-type MPM cells, when cultured *in vitro* as three-dimensional (3D) multicellular spheroids, but not when grown in conventional two-dimensional (2D) monolayers.

Consistent evidence suggests that preclinical models based on standard 2D culture of cancer cells largely fail to predict drug efficacy, because they do not recapitulate the three-dimensional architecture, heterogeneity and complexity of human tumors (44). Multicellular tumor spheroids formed from established tumor cell lines, approximating physiologic conditions, may contribute to bridging this gap.

We show by immunofluorescence that EPZ-6438 treatment nearly abolished H3K27 trimethylation, which was spatially restricted to only the external oxygenated region of the spheroid. In agreement with this, we have previously demonstrated that expression of the H3K27 demethylase KDM6B was mostly restricted to the internal hypoxic core of MPM MCS, enriched in HIF2α expression (31).

**Figure 6:**
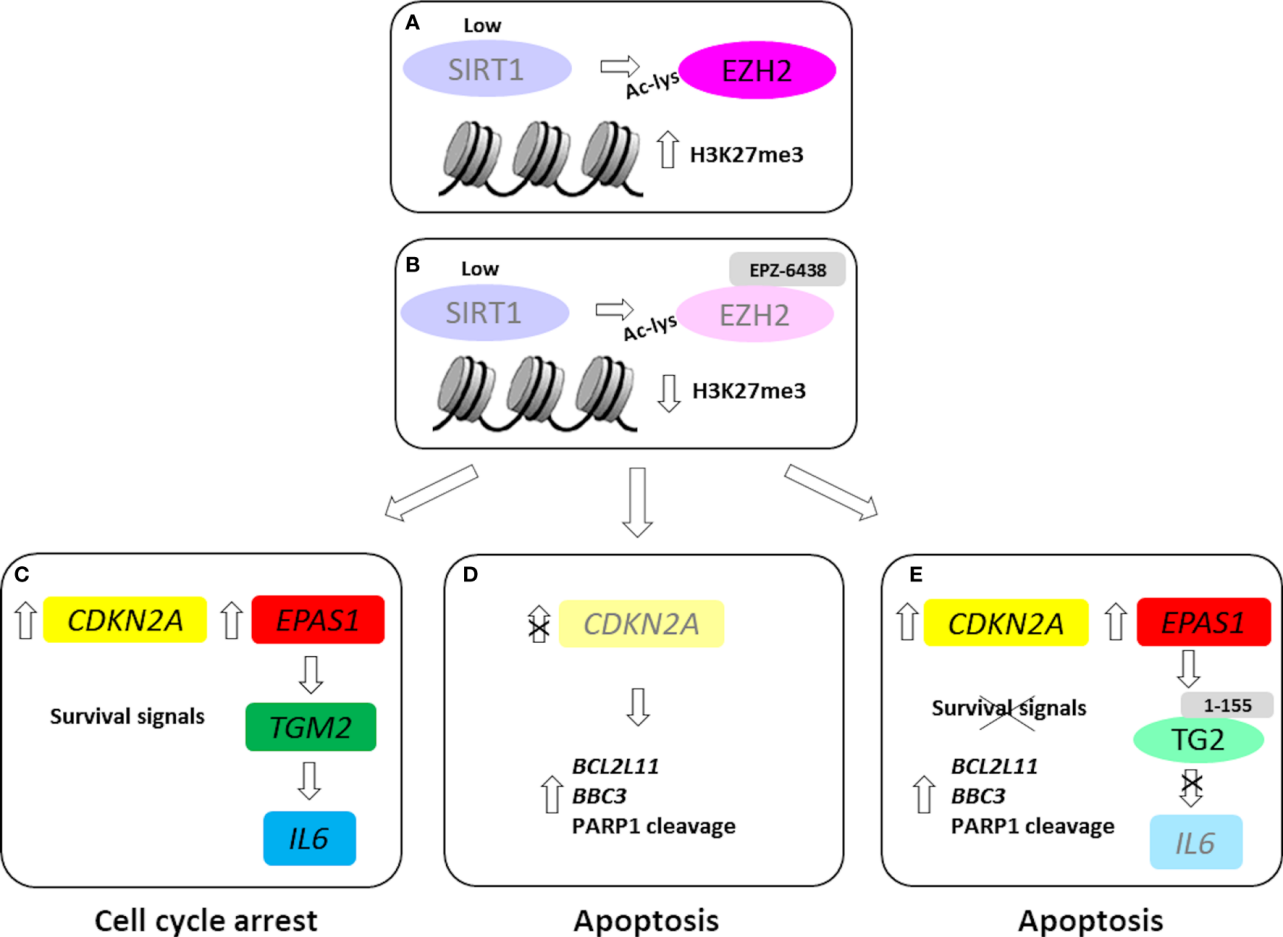
EZH2 inhibition in wild-type and *CDKN2A* silenced MPM MCS. In our MPM model, SIRT1 inhibition/silencing induced EZH2 acetylation and stability **(A)**. EZH2 inhibition, by EPZ-6438 treatment **(B)**, induced the expression *CDKN2A*, causing cell growth arrest. Furthermore, EPZ-6438 treatment upregulated *EPAS1* and downstream *TGM2* and *IL6* **(C)**. EPZ-6438 treatment induced apoptosis in *CDKN2A* silenced MPM MCS **(D)**. Combined treatment with EPZ-6438 and 1-155, a selective TG2 inhibitor, abolished *IL6* induction and led to apoptotic death **(E)**. Light colors were used to indicate low or null levels of gene expression or protein activity.

It has been described that, under metabolic or hypoxic stress, changes in NAD+/NADH are key events that initiate the cellular adaptation processes. The NAD^+^ level and NAD^+^/NADH ratio decrease during hypoxia and this is associated with SIRT1 downregulation and increased acetylation (45). We observed that SIRT1 silencing or inhibition, with EX-527, resulted in increased EZH2 protein acetylation and stability. In accordance with increased EZH2 protein expression, we observed increased H3K27me3 levels. It has been described that acetylated EZH2 exerts a gain-of-function in cells and promotes lung cancer progression (19). Interestingly, the observed highest levels of global H3K27me3 in MPM cells might indicate a higher degree of addiction towards EZH2 activity and explain increased sensitivity to its inhibition (**Figures 6A, B**).

In low SIRT1 conditions, EPZ-6438 treatment significantly reduced MPM cell proliferation, arresting cells in G0/G1 phase. We observed that EPZ-6438 treatment significantly reduced the size and modified the architecture of MPM MCS in which *SIRT1* was expressed at lower and *EZH2* at higher levels, compared to the cells cultured in 2D. Notably, in EPZ-6438 treated MCS, we observed a strong induction of *CDKN2A*, encoding for the cell cycle inhibitor p16^ink4a^. p16^ink4a^ exerts tumor suppressive function by inhibiting the activities of the cyclin D-dependent kinases, CDK4 and CDK6, and preventing the retinoblastoma protein (Rb) phosphorylation and dissociation from the transcription factor E2F1. This biochemical pathway, essential for the transition from G1 to S phase, is frequently disrupted in tumor cells, by either deletions or inactivating mutations. Furthermore, the literature supports that H3K27me3 regulates *CDKN2A* expression and that EZH2 repression of *CDKN2A* is a critical control point to promote transformation (46).

It has been described that several stress stimuli may activate p16^ink4a^ expression to orchestrate cell cycle exit and senescence response. Cellular senescence is a potent tumor suppressor mechanism, acting in coordination with the immune system to clear potentially malignant cells from the tissues. However, senescence and cancer might be considered related endpoints of accumulating cellular damage (24).

*CDKN2A* induction and cell cycle arrest were reverted upon EPZ-6438 removal, indicating growth inhibition and not cytotoxicity in cells exposed to the treatment. Indeed, our results show that MPM cells in which *CDKN2A*/p16^ink4a^ was induced were more resistant to apoptosis than *CDKN2A* silenced cells (**Figure 6C**).

Several studies have since demonstrated the reversibility of full-featured senescence, indicating that pre-senescent or early senescent cells are poised to re-enter the cell cycle. Based on the massive epigenetic remodeling underlying the state switch, senescence has also been linked to enhanced plasticity and reprogramming (47). Our data strongly suggests that EPZ-6438 treatment not only suppressed MPM cell proliferation, but also induced pro-survival pathways, which protected cancer cells from apoptosis. EZH2 inhibition increased the expression of TG2 coding gene, *via* HIF2α (31), which in turn increased the expression of the *IL-6* gene coding for the pro-inflammatory cytokine, IL-6. IL-6 has emerged as a mediator of pivotal processes in MPM, such as cell proliferation and chemoresistance (48). In MPM MCS expressing *CDKN2A*, blocking TG2 activity, by the selective inhibitor 1-155, abrogated the EPZ-6438-mediated increase in *IL-6* expression and induced apoptosis (**Figure 6E**). While, in *CDKN2A* silenced MPM MCS, EPZ-6438 treatment induced apoptosis (**Figure 6D**), probably by promoting the pro-apoptotic activity of E2F1 (49). However, the mechanistic difference between wild-type and *CDKN2A* silenced MCS response to EPZ-6438 treatment needs further and deeper investigation.

Our data support the view that treatment approaches based on inducing p16^ink4a^ and related G0/G1 growth arrest, in tumor cells that lack p16^ink4a^, can induce tumor stabilization, but may also enhance pro-survival signals and thereby confer resistance to chemotherapy treatments. Deletion of *CDKN2A* on chromosome 9p21 is a common molecular alteration in MPM. However, fluorescent *in situ* hybridization (FISH) on MPM tumor tissues has revealed that the *CDKN2A* homozygous deletion cannot be detected in all cells. Indeed, the status of the *CDKN2A* gene is highly variable, with no loss, hemizygous and homozygous losses within the same tumor. This detection of non-homogeneous deletions of *CDKN2A* suggests that besides the polyclonal origin, several genetic subclones might also exist within one tumor (8).

In conclusion, we demonstrated that, in cells with no loss or hemizygous deletion, such as the MSTO-211H MPM cells, *CDKN2A* expression can be epigenetically modulated. Translating our findings into the clinical context, we could suggest that patients with homozygous deletion for *CDKN2A* should respond to EZH2 inhibition (apoptosis). Conversely, in MPMs with wild-type *CDKN2A*, a cell growth arrested phenotype would be anticipated. Apoptosis could be augmented in a CDKN2A wild-type setting by TG2 inhibition. In conclusion, these findings open up possibilities for refining patient stratification and/or potentiation of EZH2 inhibitors.

## Data Availability Statement

The raw data supporting the conclusions of this article will be made available by the authors, without undue reservation.

## Ethics Statement

MPM tissue samples were collected in accordance with Ospedale Maggiore della Carità (Novara, Italy) approval and with patients’ informed consent.

## Author Contributions

Conception and Design: GP and LM. Data Acquisition: GP, ZW, CB, SM, DT, and RB. Analysis of Data: GP, ZW, RB, MG, DF, and LM. Writing: GP, LM, MG, and DF. All authors contributed to the article and approved the submitted version.

## Funding

The authors acknowledge the financial support of project HERMES (HEreditary Risk in MESothelioma) and Università del Piemonte Orientale (Bando ricerca locale 2019).

## Conflict of Interest

The authors declare that the research was conducted in the absence of any commercial or financial relationships that could be construed as a potential conflict of interest.
