# Corrigendum: *CDKN2A* determines mesothelioma cell fate to EZH2 inhibition

**DOI:** 10.3389/fonc.2022.1081632

**Published:** 2022-11-23

**Authors:** Giulia Pinton, Zhuo Wang, Cecilia Balzano, Sara Missaglia, Daniela Tavian, Renzo Boldorini, Dean A. Fennell, Martin Griffin, Laura Moro

**Affiliations:** ^1^ Department of Pharmaceutical Sciences, University of Piemonte Orientale (UPO), Novara, Italy; ^2^ School of Life and Health Sciences, Aston University, Birmingham, United Kingdom; ^3^ Laboratory of Cellular Biochemistry and Molecular Biology, Centro di Ricerca in Biochimica E Nutrizione dello Sport (CRIBENS), Catholic University of the Sacred Heart, Milan, Italy; ^4^ Department of Health Science, University of Piemonte Orientale (UPO), Novara, Italy; ^5^ Leicester Cancer Research Centre, University of Leicester, Leicester, United Kingdom

**Keywords:** malignant pleural mesothelioma, EZH2 inhibitor, *CDKN2A*/p16^ink4a^, TG2, multicellular spheroids

In the original article, there was an error in the Funding statement as published. The correct funding statement is “The authors acknowledge the financial support of project HERMES (HEreditary Risk in MESothelioma) and Università del Piemonte Orientale (Bando ricerca locale 2019)”.

The authors apologize for this error and state that this does not change the scientific conclusions of the article in any way. The original article has been updated.

## Publisher’s note

All claims expressed in this article are solely those of the authors and do not necessarily represent those of their affiliated organizations, or those of the publisher, the editors and the reviewers. Any product that may be evaluated in this article, or claim that may be made by its manufacturer, is not guaranteed or endorsed by the publisher.

