# Supplementary material for: CDKN2A Determines Mesothelioma Cell Fate to EZH2 Inhibition
Source: Front Oncol. 2021 Jul 1;11:678447. doi: 10.3389/fonc.2021.678447 (PMC8281343; doi:10.3389/fonc.2021.678447)
Supplement: Supplementary file 1 [file DataSheet_1.pdf]

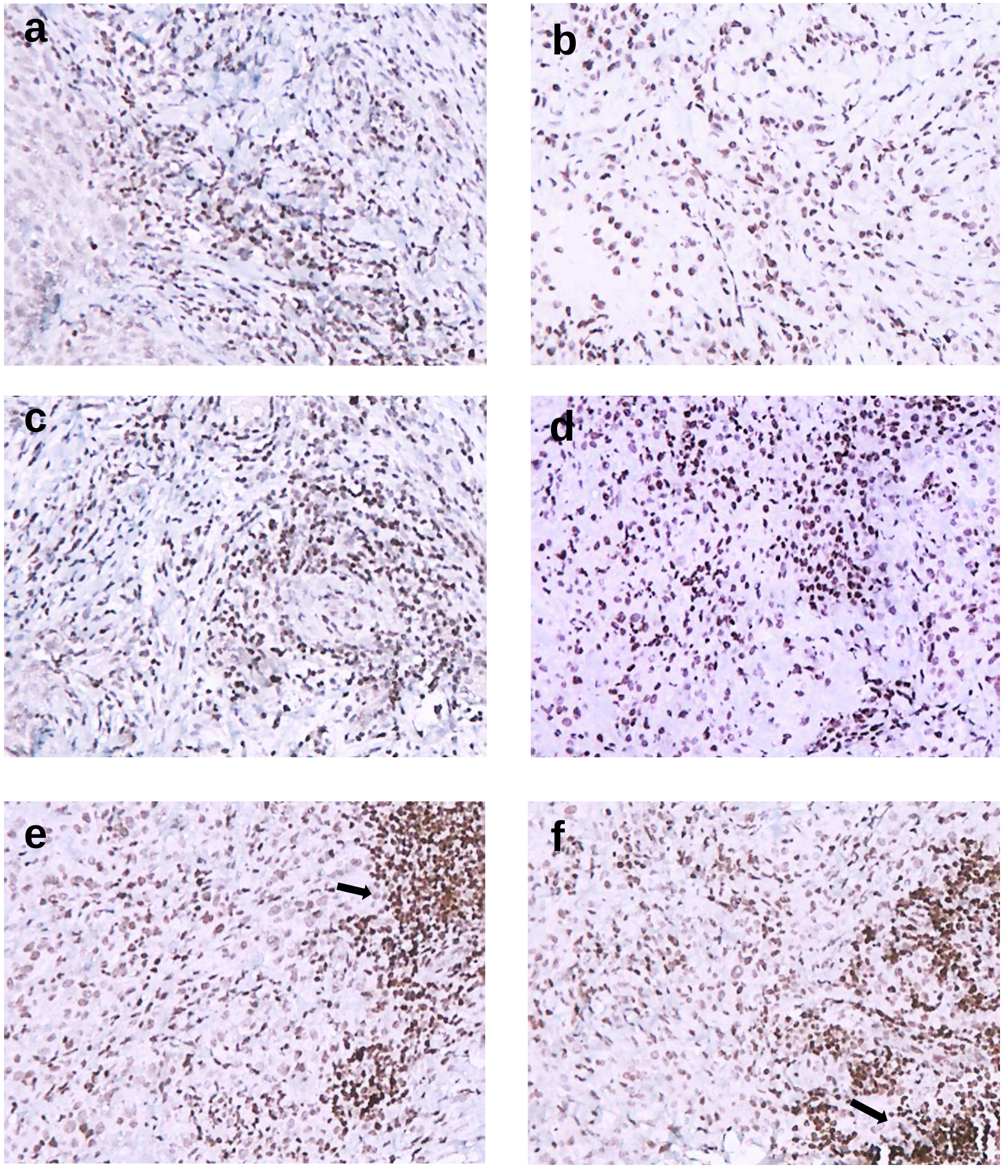

Figure S1

a-f) Representative images of immunostaining. Tumor biopsies from 6 patients diagnosed with $BAP1^+$ biphasic MPM were stained for H3K27me3. Images were captured at magnification x40 using light microscopy. Arrows indicate highly positive infiltrating inflammatory cells.

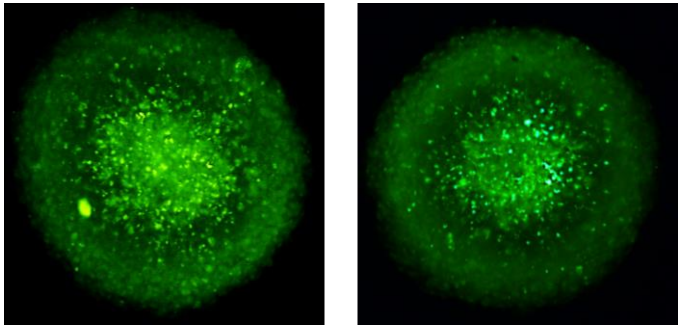

Figure S2
Representative images of immunofluorescence. MSTO-211H spheroids were stained for HIF2 and KDM6B. Images were captured using fluorescence microscopy (x40 magnification).

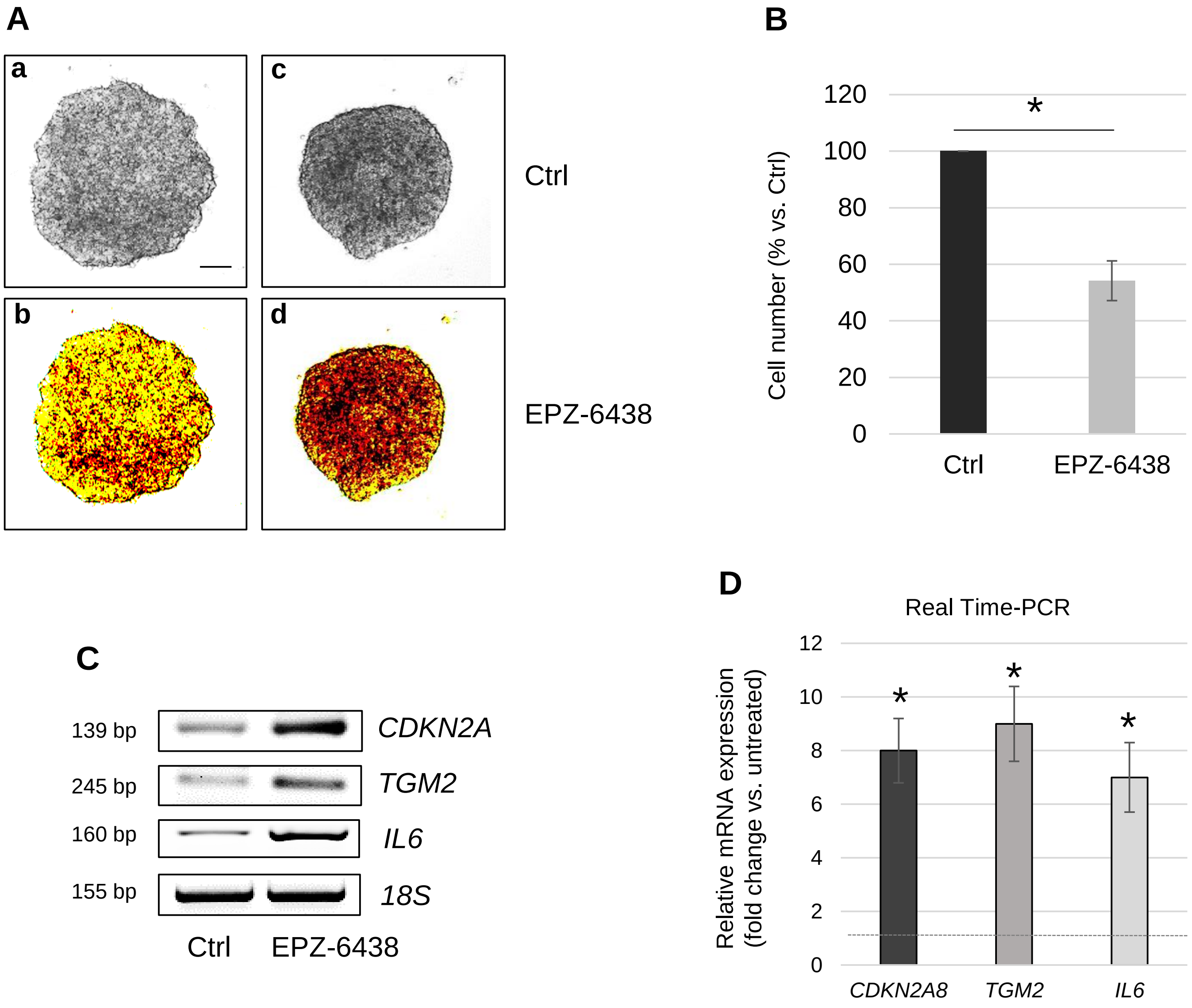

Figure S3

A) Representative phase contrast images (x40 magnification) (a,c) and relative pseudo-color images (b,d) of BR95 multicellular spheroids treated for 48 hours, with or without EPZ-6438. **B)** Bar graph shows the percentage of viability of BR95 cells cultured as monolayer (2D) ± 48 hours treatment with EPZ-6438. Each bar represents mean of three independent experiments ± s.d.,*p ≤ 0.05. **C)** Representative RT-PCR and Real Time PCR **D)** analysis of *CDKN2A*, *TGM2*, and *IL6* mRNA expression in BR95 spheroids treated or not 48 hours with EPZ-6438. 18S rRNA was used as housekeeping gene,*p ≤ 0.05.
